# Different Visualizations Cause Different Strategies When Dealing With Bayesian Situations

**DOI:** 10.3389/fpsyg.2020.01897

**Published:** 2020-08-21

**Authors:** Andreas Eichler, Katharina Böcherer-Linder, Markus Vogel

**Affiliations:** ^1^Institute of Mathematics, University of Kassel, Kassel, Germany; ^2^Institute of Mathematics, University of Freiburg, Freiburg, Germany; ^3^Institute of Mathematics and Informatics, University of Education Heidelberg, Heidelberg, Germany

**Keywords:** Bayesian reasoning, Bayesian situations, natural frequencies, strategies, visualization

## Abstract

People often struggle with Bayesian reasoning. However, previous research showed that people’s performance (and rationality) can be supported by the way the statistical information is represented. First, research showed that using natural frequencies instead of probabilities as the format of statistical information significantly increases people’s performance in Bayesian situations. Second, research also revealed that people’s performance increases through using visualization. We have built our paper on existing research in this field. Our main aim was to analyze people’s strategies in Bayesian situations that are erroneous even though statistical information is represented as natural frequencies and visualizations. In particular, we compared two pairs of visualization with similar numerical information (tree diagram vs. unit square, and double-tree diagram vs. 2 × 2-table) concerning their impact on people’s erroneous strategies in Bayesian situations. For this aim, we conducted an experiment with 540 university students. The students were randomly assigned to four conditions defined by the four different visualizations of statistical information. The students were asked to indicate a fraction in response to four Bayesian situations. We documented the numerator and denominator of the students’ responses representing a basic set and a subset in a Bayesian situation. Our results showed that people’s erroneous strategies are highly dependent on visualization. A central finding was that the visualization’s characteristic of making the nested-sets structure of a Bayesian situation transparent has a facilitating effect on people’s Bayesian reasoning. For example, compared to the unit square, a tree diagram does not explicitly visualize the set-subset relations that are relevant in a Bayesian situation. Accordingly, compared to a unit square, a tree diagram partly hinders people in finding the correct denominator in a Bayesian situation, and, in particular, triggers selecting a wrong numerator. By analyzing people’s erroneous strategies in Bayesian situations, we contribute to investigating approaches to facilitate Bayesian reasoning and to further develop the teaching of Bayesian reasoning.

## Introduction

**FIGURE 1:**
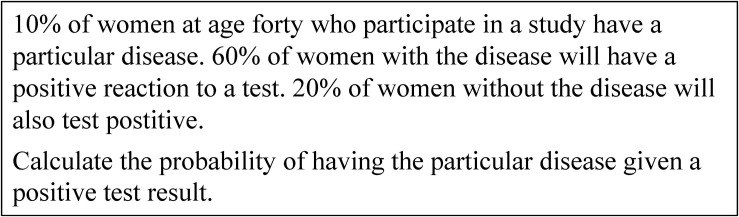
A typical Bayesian situation in an unspecific medical context (Johnson and Tubau, 2015, p. 3).

Bayes’ formula is one of the main models for dealing with inferential judgment of situations of uncertainty (Gigerenzer and Hoffrage, 1995). Reasoning in such situations, known as Bayesian situations, is a challenge for students in school (e.g., Wassner, 2004; Weber et al., 2018); adult laymen in real life (e.g., Colomé et al., 2018); and even experts in different professions, such as physicians, lawyers, or managers (Gigerenzer, 2014; Hoffrage et al., 2015). A typical Bayesian situation concerning an unspecific medical context is given in Figure 1.

Although it is important to judge Bayesian situations in various aspects of real life, research from recent decades showed that experts as well as laymen and students have severe difficulties with Bayesian reasoning (Kahneman et al., 1982; McDowell and Jacobs, 2017). McDowell and Jacobs (2017) revealed that only about 4% of people were able to calculate a probability in a Bayesian situation when the statistical information was given by percentages or rather probabilities, such as $P\,\left(\mathrm{disease}|\mathrm{test}\,\mathrm{positive}\right)=\frac{10\%\cdot 60\%}{10\%\cdot 60\%+90\%\cdot 20\%}=25\%$ representing the solution of the Bayesian situation in Figure 1.

**FIGURE 2:**
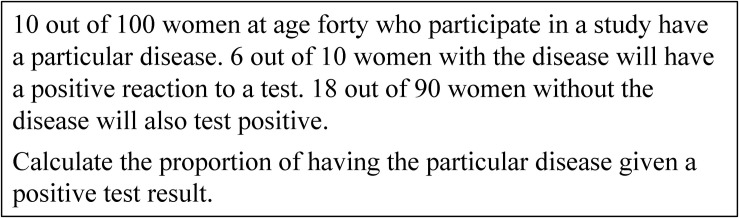
The Bayesian situation of Figure 1 with natural frequencies.

However, research gained results refer to two approaches of representing statistical information that facilitate Bayesian reasoning. Research showed that using an appropriate Bayesian strategy in a Bayesian situation is highly dependent on the way the statistical information is presented. The first approach is using natural frequencies (Gigerenzer and Hoffrage, 1995; Cosmides and Tooby, 1996). The meta-analysis by McDowell and Jacobs (2017) showed that the rate of correct responses increases from approximately 4% to about 25% if the statistical information in a Bayesian situation is presented in the form of natural frequencies. Figure 2 presents the Bayesian situation in Figure 1 using natural frequencies. The second facilitating approach is using visualization (McDowell and Jacobs, 2017). Research demonstrates a facilitating effect of different kinds of visualizations, such as tree diagrams (e.g., Sedlmeier and Gigerenzer, 2001), double-tree diagrams (e.g., Böcherer-Linder and Eichler, 2019), 2 × 2-tables (e.g., Binder et al., 2015), unit squares (e.g., Böcherer-Linder and Eichler, 2017), Euler diagrams (e.g., Sloman et al., 2003), roulette-wheel diagrams (e.g., Yamagishi, 2003), bar graphs (e.g., Starns et al., 2019), frequency grids (e.g., Sedlmeier and Gigerenzer, 2001), or icon arrays (e.g., Brase, 2009). In particular, studies using visualization in addition to natural frequencies reported an increase of correct responses in Bayesian situations from about 40–70% (Garcia-Retamero and Hoffrage, 2013; Binder et al., 2015; Böcherer-Linder and Eichler, 2017).

The aim of this paper is to contribute to the field of facilitating Bayesian reasoning by focusing on those people who fail to use the correct Bayesian strategy (Zhu and Gigerenzer, 2006) in a Bayesian situation although the statistical information is given by natural frequencies and by visualization. For this purpose, we investigate particularly erroneous and non-Bayesian strategies (cf. Zhu and Gigerenzer, 2006) of 540 undergraduate students concerning four Bayesian situations. Furthermore, we investigate relationships between erroneous strategies and properties using two pairs of visualizations of Bayesian situations. We restrict our focus to these two pairs of visualizations for two reasons. First, our aim was to investigate visualizations that are appropriate for training, regardless of available tools such as paper and pencil, or computers (cf. Bruckmaier et al., 2019). This excludes visualization representing a frequency style (Khan et al., 2015) from our study. For example, to draw an icon array with 1,000 icons is not appropriate in a paper-pencil situation. Second, from the other two styles (Khan et al., 2015), that is, the branch style and the nested style, we selected two visualizations each that were found to have a facilitating effect, but that differed in the numerical information. Thus, we investigated relationships between two pairs of visualizations, providing mostly the same numerical information (i.e., tree diagram vs. unit square, and double tree diagram vs. 2 × 2-table), and the erroneous strategies of the students. Since the main aim of our study was to investigate erroneous non-Bayesian strategies when Bayesian situations are presented in a supportive way including both natural frequencies and visualizations (cf. McDowell and Jacobs, 2017), we desisted from defining a condition in which the Bayesian situations were only supported by natural frequencies, or in which the Bayesian situations were given in a probability format. A related investigation was presented by Gigerenzer and Hoffrage (1995) or Zhu and Gigerenzer (2006).

## Theoretical Perspectives on Natural Frequencies and Visualization

Two perspectives are proposed to explain the “natural frequency facilitation effect” (McDowell and Jacobs, 2017, p. 5). The first perspective refers to an ecological rationality (Gigerenzer and Hoffrage, 1995; Johnson and Tubau, 2015). A human strategy is “ecologically rational to the degree that it is adapted to the structure of an environment” (Todd and Gigerenzer, 2000, p. 730). A possible evolutionary reason for the ecological rationality of a frequency format is “that the mind is tuned to frequency formats, which is the information format humans encountered long before the advent of probability theory” (Gigerenzer and Hoffrage, 1995, p. 697). This evolutionary explanation of the benefit of representing Bayesian situation in a frequency format was also supported by Cosmides and Tooby (1996). Gigerenzer and Hoffrage (1995) further emphasized the match between natural frequencies and a natural sampling process that leads to reduced computational complexity in a Bayesian situation (Brase and Hill, 2015; Johnson and Tubau, 2015; McDowell and Jacobs, 2017).

The second perspective is called “nested-set hypothesis” (Sloman et al., 2003, p. 297). This hypothesis is based on a dual-process model, including a “primitive” and error-prone associative system, and a rule-based system respecting the “logic of set inclusion” (Barbey and Sloman, 2007, p. 244). Thus, in this perspective, the main assumption is that a representation of statistical information that “makes nested set relations transparent” (Barbey and Sloman, 2007) triggers a rule-based system and therefore facilitates Bayesian reasoning. Accordingly, proponents of the nested-sets perspective suggest that “any manipulation that increases the transparency of the nested-sets relation should increase correct responding” (Sloman et al., 2003, p. 302; cf. also Mandel, 2015; Mandel and Navarrete, 2015). We discuss a concrete example of a transparency of nested-sets relations in visualizations in the next section.

Some researchers recommend neglecting the differences of the two theoretical perspectives on the natural frequency facilitation effect (Brase and Hill, 2015; Johnson and Tubau, 2015; McDowell and Jacobs, 2017). Thus, Johnson and Tubau (2015, p. 5) suggested that “in order to advance the discussion, we need to move away from the standard ‘natural frequency vs. nested-sets’ debate.” Putting this debate in the background means to focus on the basis of the natural frequency facilitating effect, that is, to provide an transparent structure of the statistical information and simpler computation compared to a probability format (Johnson and Tubau, 2015; McDowell and Jacobs, 2017).

There is a broad consensus that visualization facilitates Bayesian reasoning (e.g., Brase, 2009; Spiegelhalter et al., 2011; Khan et al., 2015; McDowell and Jacobs, 2017). Depending on the theoretical perspectives outlined above, different facilitating properties of visualizations are proposed. Proponents of the ecological rationality perspective suggest “real, discrete, and countable” objects as facilitating property of visualization (Cosmides and Tooby, 1996, p. 33; cf. also Tubau et al., 2019). Proponents of the nested-sets perspective suggest that “the transparency of the nested-sets” (Sloman et al., 2003, p. 302) facilitates Bayesian reasoning. Transparency means making “set inclusion and set membership” visible (McDowell and Jacobs, 2017, p. 6; cf. also Sloman et al., 2003). Accordingly, an Euler diagram or a roulette wheel diagram (Yamagishi, 2003) that include transparency of a nested-sets relation are proposed as facilitating visualization. Moro et al. (2011) also recommend making the relative proportions of sets and subsets transparent. Beyond the theoretical perspectives, Garcia-Retamero and Hoffrage (2013) or Binder et al. (2015) give evidence that visualizations have an additional facilitating effect when the statistical information in a Bayesian situation is given by natural frequencies. Our own research (Böcherer-Linder and Eichler, 2019) focused on the effect of five visualizations including the natural frequency format (tree diagram, double tree diagram, 2 × 2-table, unit square, and icon array) on people’s performance concerning Bayesian reasoning tasks. The results provided evidence that visualizing discrete and countable objects (cf. Cosmides and Tooby, 1996; Brase, 2009), and making the nested-sets relation transparent (Sloman et al., 2003; Barbey and Sloman, 2007), have a facilitating effect on people’s performance concerning Bayesian reasoning tasks. However, we found that making nested sets transparent has a much stronger effect compared to visualizing discrete and countable objects (Böcherer-Linder and Eichler, 2019).

## Visualization of Bayesian Situations

**FIGURE 3:**
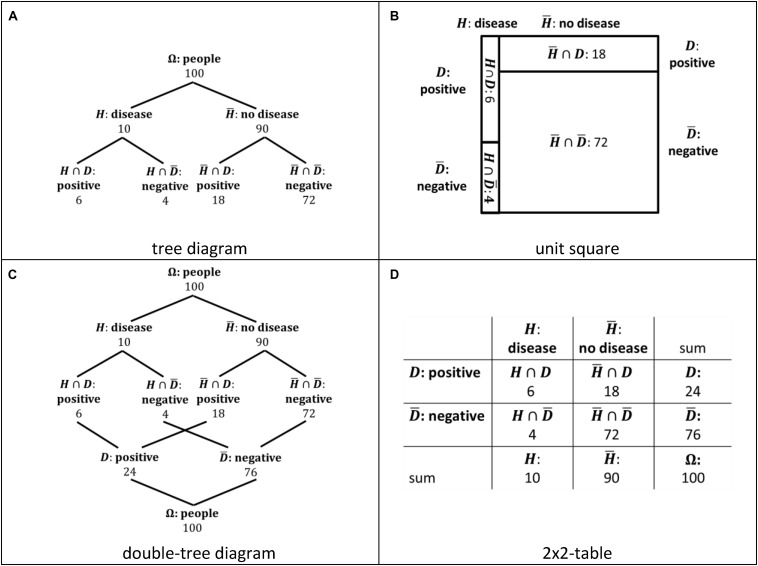
Tree diagramm **(A)**, unit square **(B)**, double-tree diagram **(C)**, and 2 × 2-table **(D)** visualizing the Bayesian situation of Figure 2. The indication of the sets were added for illustrating the discussion in the text.

This paper is based on the theoretical discussion summarized above and on existing empirical research including our own findings. Instead of comparing performance rates for Bayesian reasoning tasks, here we focus on erroneous “non-Bayesian strategies” (Zhu and Gigerenzer, 2006, p. 296) that people use instead of a correct Bayesian strategy and ask for specific characteristics of visualizations that trigger erroneous strategies. As outlined in the introduction, we restrict our focus in this research to two pairs of visualizations: tree diagram and unit square, double-tree diagram and 2 × 2-table (Figure 3). We discuss each of the four visualizations of Bayesian situations regarding their main properties below. We further refer to the solution in the medical context given in Figures 1, 2, respectively. Using the abbreviation *Ω* for a sample, *H* for hypothesis (in this case having a disease), and *D* for data (in this case a positive test result), the solution for the medical context given with natural frequencies is $P\,\left(H|D\right)=\frac{P\,\left(H\cap D\right)}{P\,\left(D\right)}=\frac{6}{6+18}=\frac{1}{4}$.

A common visualization of Bayesian situations representing a branch style (Khan et al., 2015) is a tree diagram (e.g., de Veaux et al., 2012; Utts and Heckard, 2015; Figure 3A), which is often found to facilitate Bayesian reasoning (Sedlmeier and Gigerenzer, 2001; Steckelberg et al., 2004; Binder et al., 2015; Budgett et al., 2016). A tree diagram implies a hierarchy of sets (events) that are highlighted by nodes (cf. Böcherer-Linder and Eichler, 2017; Bruckmaier et al., 2019). Thus, a set inclusion following this hierarchy, such as (*H*∩*D*)⊆*H*, is transparent (cf. also the findings of Bruckmaier et al., 2019). Concerning the solution *P*(*H*|*D*) of a Bayesian reasoning task, the set *H*∩*D* is given by a single node, but the set *D* is given by two nodes representing *H*∩*D* and $\bar{H}\cap D$. Since the nodes and the related branches are parts of different paths of the tree, the set inclusion (*H*∩*D*)⊆*D* and $\left(\bar{H}\cap D\right)\subseteq D$ is not transparent (Böcherer-Linder and Eichler, 2017). Furthermore, the hierarchy of the tree diagram implies the conjunction of events, such as *H*∩*D*, only implicitly in the second level of the tree.

A unit square (Eichler and Vogel, 2015; Figure 3B) representing a nested style (Khan et al., 2015) was also found to facilitate Bayesian reasoning (Oldford, 2003; Böcherer-Linder and Eichler, 2017, 2019; Talboy and Schneider, 2017). In a unit square, the set inclusion (*H*∩*D*)⊆*D* and $\left(\bar{H}\cap D\right)\subseteq D$ as well as (*H*∩*D*)⊆*H* and $\left(H\cap \bar{D}\right)\subseteq H$ are presented in one row or in one column. Thus, physically neighboring fields in a column or row represent subsets of the same set. For this reason, we understand a unit square as a visualization that makes the nested-sets relation in a Bayesian situation transparent. More specifically, we call this transparency “graphical transparency.” A unit square further shows the proportions of sets and subsets (cf. Moro et al., 2011). Although Talboy and Schneider (2017) suggest this area proportionality as an important property of a visualization of a Bayesian situation, we did not found a facilitating effect of this property concerning people’s performance in Bayesian reasoning tasks (Böcherer-Linder and Eichler, 2019). A unit square does not include a hierarchy. A unit square includes similar numerical information as a tree diagram concerning a Bayesian situation. We call the amount of numerical information “numerical transparency.” Although there are slight differences, we understand the numerical transparency of a tree diagram and a unit square as comparable.

A double-tree diagram (Figure 3C) has also been found to facilitate Bayesian reasoning (Wassner, 2004; Böcherer-Linder and Eichler, 2019). The double-tree diagram represents a branch style (Khan et al., 2015), and emphasizes two different hierarchies in a Bayesian situation. One hierarchy is the same as in a tree diagram, showing, for example, the relation Ω⊇*H*⊇(*H*∩*D*) with $\text{Ω}=\left(H\cup \bar{H}\right)$. The second hierarchy shows inversely, for example, the relation (*H*∩*D*)⊆*D*⊆Ω with $\text{Ω}=\left(D\cup \bar{D}\right)$. For this reason, the set inclusion (*H*∩*D*)⊆*D* and $\left(\bar{H}\cap D\right)\subseteq D$ is visualized in both cases by a branch that connects the subset with the basic set (Figure 3C). Thus, the set inclusion is transparent. In addition, a double tree diagram includes more numerical information compared to a tree diagram and a unit square, namely for every nine sets and subsets in a simple Bayesian situation, such as the situation shown in Figure 1. Thus, the numerical transparency of a double tree diagram is higher than the numerical transparency of a tree diagram and a unit square. The conjunction of events (e.g., *H*∩*D*) is visible since there exist branches to each of the two basic sets, that is, to *H* and to *D*. However, the conjunction of events is not explicitly visualized.

Further, a 2 × 2-table (Figure 3D) representing a nested style (Khan et al., 2015) facilitates Bayesian reasoning (Binder et al., 2015; Böcherer-Linder and Eichler, 2019). A 2 × 2-table includes the same numerical information of the nine sets and subsets in a simple Bayesian situation as a double tree diagram. Thus, a 2 × 2-table provides the same numerical transparency than a double tree diagram, but shows a higher numerical transparency than a tree diagram and a unit square. The set inclusion (*H*∩*D*)⊆*D* and $\left(\bar{H}\cap D\right)\subseteq D$ as well as (*H*∩*D*)⊆*H* and $\left(H\cap \bar{D}\right)\subseteq H$ is presented in one row or in one column in a 2 × 2-table. Subsets of the same set are given in neighboring fields in the same row or same column (c.f. Figure 3D; Böcherer-Linder and Eichler, 2019). For example, *H*∩*D* and $\bar{H}\cap D$ are represented by neighboring fields in the same row in a 2 × 2-table. A 2 × 2-table does not include a hierarchy of events. The conjunction of events such as *H*∩*D* is explicitly visualized. For example, the events H and D are represented by a side of a field that represents the conjunctive event *H*∩*D*.

To conclude, if a set and subset are connected by a branch (or path) or are given by neighboring fields in a row or column, we assume the transparency of a set inclusion and, thus, the transparency of a set-subset relation in a Bayesian situation (graphical transparency). Furthermore, a visible relation between two sets and their intersection set makes the nested-sets structure of a Bayesian situation transparent (cf. Barbey and Sloman, 2007; McDowell and Jacobs, 2017). Finally, we differentiated between the two pairs of visualizations concerning the amount of numerical information (numerical transparency). A tree diagram and a unit square provide mostly the same numerical information, although there are slight differences. For example, in a tree diagram, there is additional numerical information of the sample size (#Ω), as compared to the unit square. The double tree diagram and the 2 × 2-table provide the same numerical information.

## Strategies in Bayesian Situations

**FIGURE 4:**
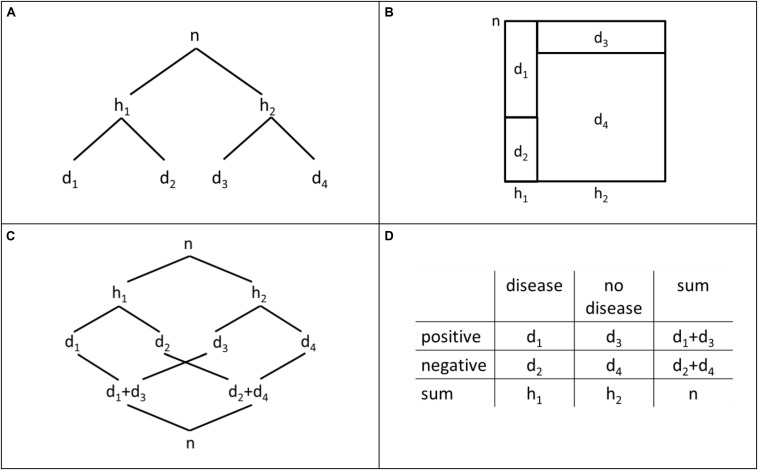
Tree diagram **(A)**, unit square **(B)**, double-tree diagram **(C)**, and 2 × 2-table **(D)** with natural frequencies.

To summarize the existing knowledge about people’s strategies in Bayesian situations, we use Figure 4, including a tree diagram, a unit square, a double-tree diagram, and a 2 × 2-table. For every visualization, *n* is the size of on abstract sample. Based on *n*, we define the following natural frequencies: $h_{1}:=n\cdot P\,\left(H\right),h_{2}:=n\cdot P\,\left(\bar{H}\right),d_{1}:=h_{1}\cdot P\,\left(D|H\right),d_{2}:=h_{1}\cdot P\,\left(\bar{D}|H\right),d_{3}:=h_{2}\cdot P\,\left(D|\bar{H}\right)$ and $d_{4}:=h_{2}\cdot P\,\left(\bar{D}|\bar{H}\right)$. A Bayesian strategy (Zhu and Gigerenzer, 2006) produces the correct response $P\,\left(H|D\right)=\frac{d_{1}}{d_{1}+d_{3}}$.

Since the correct identification of the basic set *D* is crucial in a Bayesian situation, we first refer to erroneous strategies involving a correct identification of the basic set *D*. After this, we report other erroneous strategies.

A strategy first described by Zhu and Gigerenzer (2006) is called “pre-Bayes” and is represented by the quotient of $\frac{h_{1}}{d_{1}+d_{3}}$. In this strategy, the correct basic set *D*, or rather, the frequency of *d*_1_ + *d*_3_, is chosen as denominator, but an incorrect numerator is chosen by confusing the sets *H* and *H*∩*D*.

The strategy is “evidence only” (Zhu and Gigerenzer, 2006), is represented by the quotient of $\frac{d_{1}+d_{3}}{n}$. In this strategy, the correct basic set, that is, $D=\left(H\cap D\right)\cup \left(\bar{H}\cup D\right)$ is connected to the whole sample (Ω) represented by the frequency of *n*.

Further strategies do not include *D*, or rather the frequency *d*_1_ + *d*_3_, but include *H*∩*D* as subset represented by d_1_ as the numerator of the correct solution. One erroneous strategy is described in mathematics education research (Diaz and Batanero, 2009) as well as in psychological research (Zhu and Gigerenzer, 2006) and is given by $\frac{d_{1}}{h_{1}}$. This strategy is based on the reciprocal value of the conditional probability of the correct Bayesian strategy. For this reason, Diaz and Batanero (2009) called this strategy “transposed conditional” fallacy. Zhu and Gigerenzer (2006) named this strategy “representative thinking” following Dawes (1986). A further name was given by Gigerenzer and Hoffrage (1995), who called this strategy “Fisherian.”

A further erroneous strategy is called “joint occurrence” and is represented by the quotient of $\frac{d_{1}}{n}$ (Zhu and Gigerenzer, 2006). In this case, people seem to over-emphasize the conjunction *H*∩*D*, and to neglect $\bar{H}\cap D$.

An erroneous strategy that neither includes the correct basic set *D* represented by the frequency *d*_1_ + *d*_3_ nor the subset *H*∩*D* represented by frequency d_1_ is called “conservatism” and is given by the quotient of $\frac{h_{1}}{n}$ (Zhu and Gigerenzer, 2006). The same strategy is called “base-rate only” by Gigerenzer and Hoffrage (1995).

Diaz and Batanero (2009) described an erroneous strategy without naming it that is represented by the reciprocal value of the correct quotient, that is, $\frac{d_{1}+d_{3}}{d_{1}}$. We call this strategy “inverse Bayes.” This strategy may be explained through correct identification of the basic set and the subset in a Bayesian situation but also through confusing the correct relationship of the frequencies representing these sets.

Further erroneous strategies were reported by Gigerenzer and Hoffrage (1995), but these strategies were restricted to a probability format of statistical information (e.g., a likelihood-subtraction). In addition, some erroneous strategies that were observed in the cited studies were not categorized since the frequency of these strategies were small. Gigerenzer and Hoffrage (1995) summarized related strategies as “not identified strategies,” Zhu and Gigerenzer (2006) subsumed these strategies to “guessing.”

A study by Bruckmaier et al. (2019) also focused on people’s strategies in Bayesian situations. Since the study was based on an eye-tracking method, the study included a very small sample size of 24 students. Bruckmaier et al. (2019) found only strategies discussed so far for the students’ Bayesian reasoning to be supported by natural frequencies and a tree diagram or 2 × 2-table. The findings concerning the tree diagram supported the hypothesis that the hierarchy of the tree diagram triggers people to identify a subset-set relation in different levels of the tree. The results referring to the 2 × 2-table are difficult to interpret for our purposes, because participants solved the same tasks with the 2 × 2-table that had been solved before with the tree diagram.

Although the participants, materials, and methods were different in the cited studies, we present the frequencies for the Bayesian strategy and further erroneous strategies for different studies and samples in Table 1.

**TABLE 1:** People’s strategies for dealing with Bayesian situations in prior research.

**Authors**	**Zhu and Gigerenzer, *n* = 135, young students**	**Gigerenzer and Hoffrage, *n* = 405, univ. students**	**Bruckmaier, Binder, Krauss and Kufner, *n* = 24, university students**		**Diaz and Batanero, *n* = 177 and 206**
				
**Format**	**Frequency**	**Frequency**	**Frequency**	**Probability**	**Probability**
**Visualization**	**None**	**None**	**Tree (2 × 2-table)**	**Tree (2 × 2-table)**	**None**
**Strategy**					
Bayesian strategy *d*_1_/(*d*_1_ + *d*_3_)	36.9%	45.8%	43.3% (81%)	29.5% (32%)	Not reported
Pre-Bayes *h*_1_/(*d*_1_ + *d*_3_)	11.5%	Not reported	2.2% (0%)	Not reported	Not reported
Evidence only (*d*_1_ + *d*_3_)/*n*	4.6%	Not reported	Not reported	10% (0%)	Not reported
Representative thinking *d*_1_/*h*_1_	1.8%	12.3%	17.4% (4.3%)	37.5% (2.1%)	Without frequency
Joint occurrence *d*_1_/*n*	Not reported	4.5%	21.7% (8.5%)	8% (55.3%)	Not reported
Conservatism, Base rate only *h*_1_/*n*	5.3%	2.9%	Not reported	Not reported	Not reported
Inverse Bayes (*d*_1_ + *d*_3_)/*d*_1_	Not reported	Not reported	Not reported	Not Reported	Without frequency
Guessing and other strategies	39.8%	33.5%	15.2% (6.4%)	15% (10.6%)	Not reported

In each of the cited studies, the focus is on strategies representing people’s way of identifying a combination of a basic set and subset, or rather, a fraction. In this study, we aim at enhancing the focus by differentiating between choosing a denominator and a numerator of a fraction representing a basic set and subset. Given the specific properties of the visualizations of Bayesian situations, we hypothesize that different visualizations trigger people to choose specific basic sets and subsets.

## Hypotheses

Our approach is to analyze which set (numerator) and subset (denominator) people choose depending on the different visualizations. Based on this, a structured set of hypotheses refers to the following selection of a denominator and numerator in a Bayesian situation:

H1: Selection of the correct denominator
  H1.1: Selection of the correct numerator provided the denominator is correct
    H1.1.1: Specific response in the numerator provided the denominator is correct
H2: Selection of the correct numerator
  H2.1: Selection of the correct denominator provided the numerator is correct
    H2.1.1/2: Specific responses in the denominator provided the numerator is correct
H3: Erroneous strategy depending on the numerical proportion of numerator and denominator

Now, we provide the rationale behind every hypothesis and formulate the hypotheses more specifically. Since we divided the four visualizations in two pairs of visualizations, in which each pair of visualization provides the same amount of numerical information (numerical transparency), we also divided the hypotheses for each pair: the hypotheses labeled “a” concern the pair of tree diagram and unit square, and the hypotheses labeled “b” concern the pair of double tree diagram and 2 × 2-table. Finally, we do not formulate directional hypotheses referring to the facilitating effect of visualizations between the two pairs of visualizations.

A main challenge in Bayesian situations is to identify the correct basic set (*D*), that is, to identify *d*_1_ + *d*_3_ (Figure 4) as the denominator in Bayes’ formula (cf. Sloman et al., 2003). In a tree diagram, the subsets *H*∩*D* and $\bar{H}\cap D$ are represented by two nodes of different paths that have no visible direct relation. Thus, the set inclusion (*H*∩*D*)⊆*D* and $\left(\bar{H}\cap D\right)\subseteq D$ is not transparent (cf. McDowell and Jacobs, 2017). To use the correct denominator *d*_1_ + *d*_3_ requires adding the two frequencies d_1_ and d_3_. In a unit square, the subsets *H*∩*D* and $\bar{H}\cap D$ are directly related since they are represented by neighboring fields (in a row). Thus, the set structure of a Bayesian situation and the set inclusion (*H*∩*D*)⊆*D* and ($\bar{H}\cap D)\subseteq D$ is more transparent than in the tree diagram (cf. Sloman et al., 2003; Moro et al., 2011). As in the tree diagram, the correct denominator in a Bayesian situation, that is, *d*_1_ + *d*_3_ has to be computed by a simple addition. For this reason, the first main hypothesis is as follows:

Hypothesis 1a: People who use a unit square refer to d_1_ + d_3_ as the denominator more frequently than those who use a tree diagram.

In a double-tree diagram, both subsets *H*∩*D* and $\bar{H}\cap D$ are connected to the basic set *D* by a branch. Thus, the set inclusion mentioned above is transparent in the hierarchy of the double-tree diagram. Further, the correct denominator in Bayes’ formula is directly given as a frequency and needs no additional computation (numerical transparency). In a 2 × 2-table, the two subsets *H*∩*D* and $\bar{H}\cap D$ are represented by neighboring fields (in a row), and the frequency of the basic set D, that is, the frequency *d*_1_ + *d*_3_, is directly given. Since the double tree diagram and unit square do not seem different regarding numerical and graphical transparency, we did not formulate a directed hypothesis.

Based on the correct identification of the basic set *D* and the denominator *d*_1_ + *d*_3_, it is a further challenge to identify the correct subset *H*∩*D*, or rather, the correct numerator *d*_1_ in Bayes’ formula (cf. Sloman et al., 2003). In the hierarchy of a tree diagram, *H*∩*D* and $H\cap \bar{D}$ appear explicitly as subsets of *H*. Moreover, $\bar{H}\cap D$ and $\bar{H}\cap \bar{D}$ appear explicitly as subsets of $\bar{H}$. However, the tree diagram does not make the set inclusion (*H*∩*D*)⊆*D* transparent since (*H*∩*D*) and $\left(H\cap \bar{D}\right)$ are not directly related. In a unit square, the set inclusion $\left(H\cap D\right)\subseteq \left(H\cap D\right)\cup \left(H\cap \bar{D}\right)$ is directly related since it is visualized by neighboring fields of a row. If the basic set D was identified before, the mentioned set inclusion is transparent. For this reason, the structure of the tree diagram seems to hinder people in identifying both the basic set and subset in a Bayesian situation. Hence, a subsequent hypothesis is as follows:

*Hypothesis 1.1a: Restricted to those who identify d_1_* + *d_3_ as correct denominator: People who use a tree diagram fail to identify d_1_ as numerator of the correct solution more frequently than those who use a unit square*.

A double-tree diagram makes this set inclusion outlined above transparent: In the second hierarchy of a double tree, the set inclusion (*H*∩*D*)⊆*D* is given by a branch. The set inclusion (*H*∩*D*)⊆*D* is also visualized in a 2 × 2-table in a row including two frequencies of subsets and the sum of these two frequencies. For this reason, we did not formulate a directed hypothesis regarding a difference between the double tree diagram and the 2 × 2-table.

People who correctly identified the basic set *D* and the related frequency *d*_1_ + *d*_3_ may fail to identify the correct numerator (*d*_1_) in Bayes’ formula. Based on our main assumption about the transparency of a set inclusion, in a tree diagram *H*, $\bar{H}$, or *Ω* are transparently related to *H*∩*D* and $\bar{H}\cap D$ (Figure 4). To differentiate between the three possible sets, we follow Zhu and Gigerenzer (2006), who argued that people do not use a combination of a numerator and a denominator that results in a fraction above 1 (cf. also Chapman and Liu, 2009). However, the mentioned fraction with a denominator *d*_1_ + *d*_3_ is below 1 only for specific numerators *h*_1_ and is never below 1 for a numerator n. The possible quotient *h_1_/(d_1_* + *d_3_)* is known as pre-Bayes strategy by Zhu and Gigerenzer (2006), but this quotient is not always below 1. Thus, the pre-Bayes strategy is highly dependent on the Bayesian situation and the concrete frequencies in this situation. This is apparent also in the results of Bruckmaier et al. (2019), who used two situations with *h_1_/(d_1_* + *d_3_)* > 1 and, accordingly, found nearly no pre-Bayes strategy. In our study, we used situations with *h_1_/(d_1_* + *d_3_)* > 1, and situations with *h_1_/(d_1_* + *d_3_)* < 1. Considering Zhu and Gigerenzer (2006), we expect few answers representing the pre-Bayes strategy in the first case. We refer later to the difference of situations concerning the value of *h_1_/(d_1_* + *d_3_)* below or above 1.

Referring to the transparency of a set-subset relation, for a unit square there is no meaningful reason to select *H*, or rather *h*_1_, as the numerator in a Bayesian situation.

A similar difference could be identified concerning the second pair of visualizations: In a double tree diagram, *H*∩*D* and $\bar{H}\cap D$ are obviously transparently related to *D* by a branch. However, *H* and $\bar{H}$ or *Ω* are related to *D* by a path (Figure 4). For this reason, the erroneous pre-Bayes strategy is also plausible for the double tree diagram if people fail to identify *d*_1_ as the correct numerator. For a 2 × 2-table there is no meaningful reason to select *H*, or rather *h*_1_, as the numerator in a Bayesian situation. Thus, our hypotheses are as follows:

*Hypothesis 1.1.1a: Restricted to those who identify d_1_* + *d_3_ as correct denominator: People who use a tree diagram use h_1_ as numerator in a Bayesian situation more frequently than those who use a unit square.*
*Hypothesis 1.1.1b: Restricted to those who identify d_1_* + *d_3_ as correct denominator: People who use a double tree diagram use h_1_ as numerator in a Bayesian situation more frequently than those who use a 2* × *2-table.*

The corpus of hypotheses formulated so far focuses on selection of the basic set (correct: *D*) in a Bayesian situation or the denominator (correct: *d*_1_ + *d*_3_) in Bayes’ formula. However, it is possible to change the perspective and focus on the selection of a subset, or rather, a numerator in a Bayesian situation. Actually, the visualizations allow for selecting a frequency representing a set, and selecting a second frequency representing either a basic set or a subset. The correct subset *H*∩*D* is transparently visualized as a conjunction of two sides, representing the sets *H* and *D* in the related field in a unit square and a 2 × 2-table. This structure of sets and the subset *H*∩*D* does not seem to be as transparent as in the double tree diagram, since *H* and *D* represent paths in two different hierarchies. The tree diagram does not make the structure of the sets *H* and *D* and the subset *H*∩*D* explicitly transparent. For this reason, we expect a unit square and 2 × 2-table to facilitate the identification of the conjunction *H*∩*D* as a relevant subset in a Bayesian situation. Thus, the second main hypothesis is as follows:

Hypothesis 2a: People who use a unit square refer to d_1_ as the numerator in the correct solution more frequently than those who use a tree diagram.
*Hypothesis 2b: People who use a 2* × *2-table refer to d_1_ as the numerator in the correct solution more frequently than those who use a double tree diagram.*

Furthermore, with the same rationale outlined for hypothesis 1.1, it is possible to develop a hypothesis based on correct selection of the subset *H*∩*D*, or rather, the correct numerator *d*_1_. The basic set *D* is not transparent in the tree diagram (see above), but is transparently visualized in a unit square. For this reason, a further hypothesis is as follows:

*Hypothesis 2.1a: Restricted to those who identify d_1_ as correct numerator: People who use a unit square refer to d_1_* + *d_3_ as the denominator in their solution more frequently than those who use a tree diagram*.

Since there is no theoretical difference concerning the numerical or graphical transparency of a double-tree diagram and a 2 × 2-table, we formulated no directional hypothesis concerning the identification of the correct denominator given a correct numerator.

With the same argumentation as outlined above, the hierarchy of a tree (and partly also the double-tree) may influence the selection of a denominator (basic set) using a path of the tree, namely *h*_1_ or *n*. Hence, a further pair of hypotheses regarding an erroneous response with the correct numerator in a Bayesian situation is as follows:

Hypothesis 2.1.1a: Restricted to those who identify d_1_ as correct numerator: People who use a tree diagram use h_1_ as denominator in a Bayesian situation more frequently than those who use a unit square.
*Hypothesis 2.1.1b: Restricted to those who identify d_1_ as correct numerator: People who use a double tree diagram use h_1_ as denominator in a Bayesian situation more frequently than those who use a 2* × *2-table.*
This confusion is called “representative thinking” strategy in Table 1.
Hypothesis 2.1.2a: Restricted to those who identify d_1_ as correct numerator: People who use a tree diagram, use n as denominator in a Bayesian situation more frequently than those who use a unit square.
*Hypothesis 2.1.2b: Restricted to those who identify d_1_ as correct numerator: People who use a double tree diagram use n as denominator in a Bayesian situation more frequently than those who use a 2* × *2-table.*
*This confusion is called “joint occurrence” strategy in Table 1*.

Referring to people’s strategies in Bayesian situations reported so far, we neglected the evidence-only strategy, that is, (*d_1_* + *d_3_)/n*, and the conservatism strategy, that is, *h_1_/n*. We analyzed both erroneous strategies without a directional hypothesis for both pairs of visualizations.

As outlined above, an erroneous strategy may highly be influenced by the given situation that is represented by specific natural frequencies. For example, if *h_1_/(d_1_* + *d_3_)* > 1, we expect only few people to use the pre-Bayes strategy compared to situations in which *h_1_/(d_1_* + *d_3_)* < 1. For this reason, we formulate – independent from specific visualizations – the following hypothesis:

*Hypothesis 3: In Bayesian situations with h_1_/(d_1_* + *d_3_) < 1, people follow a pre-Bayes strategy more frequently compared to Bayesian situations with h_1_/(d_1_* + *d_3_) > 1*.

## Materials and Methods

Our sample consisted of 540 undergraduate students enrolled in two mathematics courses for prospective primary school teachers. Bayesian reasoning was not part of their curriculum.

The students were randomly assigned to the four visualizations. The subsamples differed a little and had the following sizes: 122 students were assigned to the tree diagram, 120 students to the double tree diagram, 146 students to a 2 × 2-table, and 152 students to a unit square.

**FIGURE 5:**
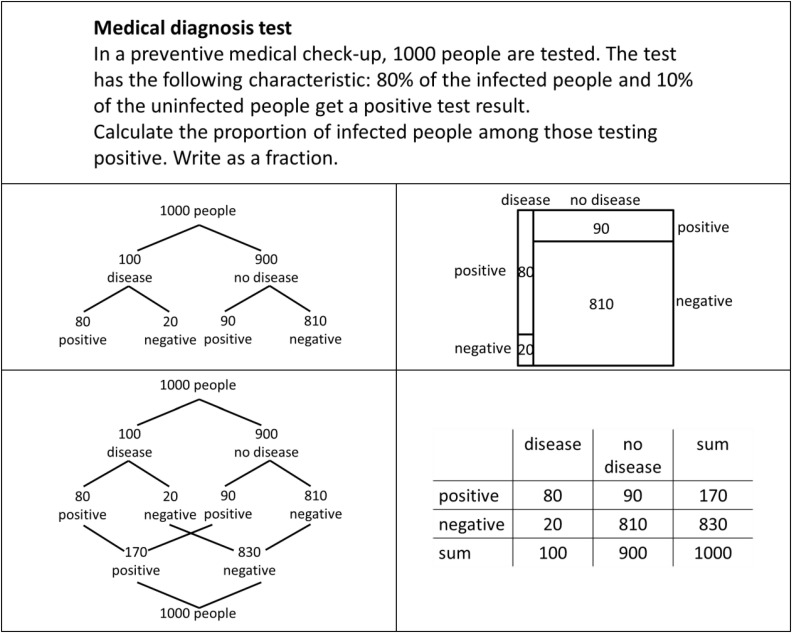
Sample task including a Bayesian situation. In the original tasks, only one of the four visualizations was shown.

Each student received a test referring to a specific visualization, such as a tree diagram, comprising two parts. The first part consisted of one page with a brief explanation of how to construct a specific visualization (cf. Böcherer-Linder and Eichler, 2017). Every explanation started with a table including the statistical information in a natural frequency format. The explanations for every visualization consisted of two further steps describing how to construct the specific diagram. The number of explanation-steps was kept constant to provide the same amount of supporting information in every condition. However, the explanations among the visualizations differed due to their different characteristics. Also, the level of familiarity was different among the visualizations. 98% of students indicated familiarity with a tree diagram, and 86% indicated familiarity with a 2 × 2-table. By contrast, only 33% were familiar with a unit square, and 28% were familiar with a double tree diagram. We discuss these differences later. The second part of the questionnaire consisted of four Bayesian tasks. One of the tasks is given in Figure 5, and the other tasks are available in a free accessible repository^1^. In these tasks, the Bayesian situation was represented by only one specific visualization. We did not use natural frequencies in the brief description of the Bayesian situation in the text (except the total sample size), but only within the visualizations. Therefore, problems could only be solved by reading the information from the visualization. This decision was made to be able to analyze the facilitating effect of the visualization. In every Bayesian situation, we asked students to indicate a fraction representing the mathematical expression for the relation of the cardinal numbers of the set (denominator) and subset (numerator). Thus, the fraction is an expression of the data partition in a Bayesian situation (Barbey and Sloman, 2007). In this regard, to ask for a fraction is the mathematical version of a single-step frequency question (Girotto and Gonzalez, 2001). Asking for a fraction is also related to the common format for responses in textbooks for school or university (e.g., Utts and Heckard, 2015).

The students had 15 min to complete the test. No intervention was delivered during the test.

The numbers in every Bayesian situation were chosen in a way that allowed identifying which sets a student had selected for determining the numerator and the denominator of his or her response. As mentioned before, the focus on the denominator and numerator allows for specifying the students’ identification of basic sets and subsets in a Bayesian situation. In some of the tasks, one of which is shown in Figure 5, the fraction *h_1_/(d_1_* + *d_3_)* is below 1; in other tasks, the fraction *h_1_/(d_1_* + *d_3_)* is above 1.

For analyzing students’ strategies, we regarded only those solutions that included a fraction or a number. There were also students who completed, for example, two tasks, but did not provide a solution to the other two tasks. For this reason, the amount of strategies that students showed differed among the four Bayesian situations. In the results section, we indicate the number of strategies shown by the students, as well as the missing responses. The data is provided in a free accessible repository (see text footnote 1).

Firstly, we documented each combination of a denominator and numerator in a descriptive way, also including versions that were cancelled down. Following Zhu and Gigerenzer (2006), we did not analyze the few solutions that provided a fraction above 1 in detail, except for the specific analysis concerning hypothesis 3. For this reason, we did not regard the inverse Bayes’ strategy that Diaz and Batanero (2009) proposed (see Table 1).

For the inferential analysis, we referred to systematic strategies. To estimate whether a student’s response represented a systematic strategy or was a result of guessing, we followed Zhu and Gigerenzer (2006) and compares the student’s responses with a guessing model. The basis of this model is the amount of single numbers and simple sums of two numbers that are provided in a Bayesian situation. Nine of these numbers or sums are given in a 2 × 2-table (Figure 3). We further added 1 as a possible number since some of the students’ responses consisted of a natural number. In these cases, we assumed a denominator of 1. We further assumed that the students chose two different numbers or sums representing different sets for the numerator or denominator. Thus, we regarded 10 × 9 = 90 different possible responses. Only half of these responses consisted of a fraction below 1. One of these responses represents the Bayesian strategy. For erroneous strategies, we assumed a uniform distribution and, accordingly, a probability of 1/44 for every strategy. We used this model to decide whether a response was based on a systematic strategy or guessing. We used a binomial distribution in which *p* equals 1/44 and *n* is given by the number of erroneous responses for a specific visualization. Based on this distribution, we determined an integer *k* for that the probability of the interval [*k*; *n*] is lower than 0.05, but bigger than 0.05 for [*k*-1; *n*]. Table 2 shows the values of *k* for the different visualizations. Thus, if a certain erroneous strategy is given in *k* or more than *k* of the students’ responses, we defined this strategy as systematic erroneous strategy.

**TABLE 2:** Limits for estimating an erroneous strategy as systematic.

**Visualization**	**Tree diagram**	**Unit square**	**Double-tree**	**2 × 2-table**
*n*	272	235	194	156
*k*	10	9	8	7

We used a χ^2^–test for independence for the statistical analyses. To measure the effect of differences between two visualizations, we used the odds ratio, but also reported Cohen’s *d*.

This experiment was carried out in accordance with the University Research Ethics Standards. Participation was voluntary, without financial incentives, and anonymity was guaranteed. A written, informed consent was not required as per local legislation and institutional requirements.

## Results

### Strategies

First, we describe the results in a descriptive way, concerning absolute and relative frequencies with which the students indicated different fractions in the four Bayesian situations. We consider these fractions by indicating the numerator and the denominator.

**FIGURE 6:**
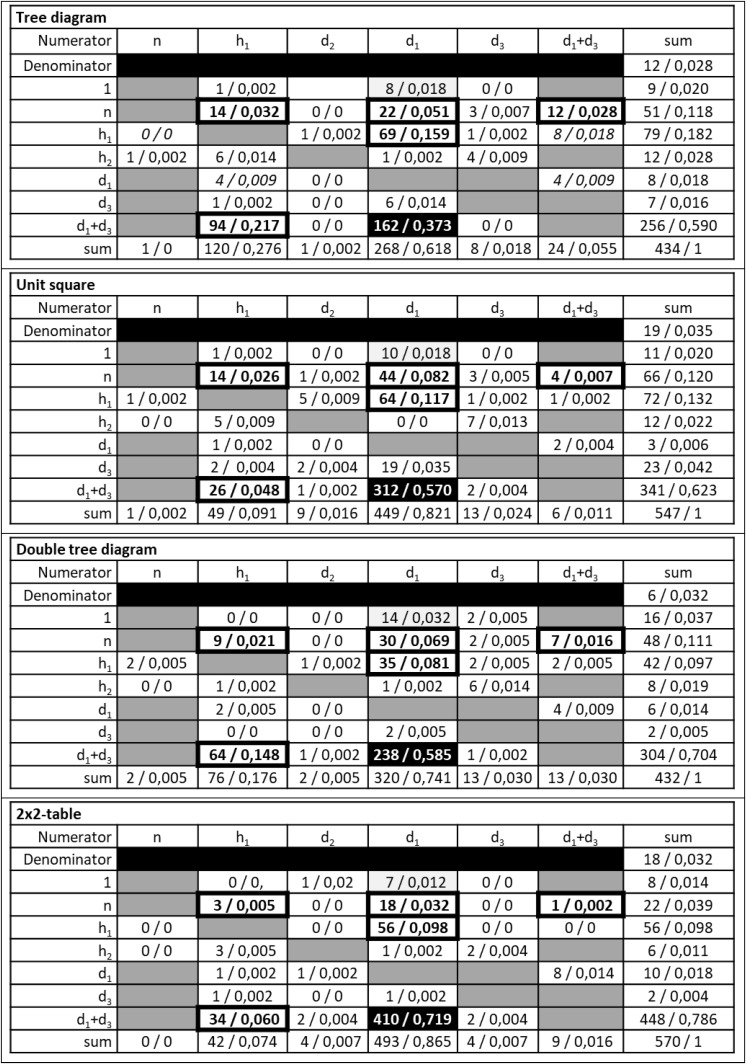
Students’ answers to Bayesian tasks differentiated to denominators and numerators.

Each table in Figure 6 shows the numerators that the students at least once provided in the first row, and the denominators that the students at least once provided in the first column. In each cell, the absolute frequency and relative frequency are given. The last row and the last column indicate the sums. The sum in the second row indicates the number of responses that could not be interpreted. The gray shaded fields represent fractions that no student provided as response. Further, the fields with a thick frame represent the fractions that were reported as an erroneous strategy in literature (cf. Table 1). The black field represents the Bayesian strategy.

The results concerning systematic strategies are given in Table 3, based on the guessing model outlined in the methods section. The strategies are sorted in the same way as in Table 1. The frequencies refer to the number of responses in which the fraction in the first column or an equivalent fraction was indicated. Beyond the erroneous strategies reported so far, we identified and labeled two further erroneous strategies with regard to existing strategies, namely, a pure evidence strategy, and a likelihood strategy. These two erroneous strategies may be understood as systematic strategies for at least one of the four visualizations, and are given in Table 3 in italics. The category “guessing” includes the amount of responses that could not be interpreted or that were seldom indicated. Finally, we indicated the amount of missing responses for every visualization. The impact of the visualization on the amount of missing responses is highly significant. Here, a very familiar visualization, a 2 × 2-table, has significantly less missing responses than the other three visualizations. However, since our aim was to analyze people’s erroneous strategies in Bayesian situations and the impact of different visualizations on these strategies, we neglect the missing responses in the following section. For an analysis of people’s performance in Bayesian situations when using visualizations that also include incomplete tasks, see Böcherer-Linder and Eichler (2019).

**TABLE 3:** Descriptive results of students’ responses concerning the Bayesian strategy and erroneous strategies. *n* indicates the number of students in a condition. The percentages are related to the amount of responses (excluding missing responses). The amount of missing responses is also given.

**Visualization**	**Tree diagram (*n* = 122)**	**Unit square (*n* = 154)**	**Double-tree (*n* = 120)**	**2 × 2-table (*n* = 148)**	**Sum average**
Bayesian strategy *d*_1_/(*d*_1_ + *d*_3_)	162/37.3%	312/57.0%	238/55.1%	410/72.4%	1122/56.7%
Pre Bayes *h*_1_/(*d*_1_ + *d*_3_)	94/21.7%	26/4.8%	64/14.8%	34/6.0%	218/11.0
Evidence only (*d*_1_ + *d*_3_)/*n*	12/2.8%	4/0.7%	7/1.6%	1/0.2%	24/1.2%
Representative thinking (*d*_1_/*h*_1_)	69/15.9%	64/10.7%	35/8.1%	56/9.9%	224/11.3%
Joint occurrence *d*_1_/*n*	22/5.1%	44/8.0%	30/6.9%	18/3.2%	114/5.2%
Conservatism *h*_1_/*n*	14/3.2%	14/2.6%	9/2.1%	3/0.5%	40/2.0%
*Pure evidence d_1_/1*	*8/1.8%*	*10/1.8%*	*14/3.2%*	*7/1.2%*	*39/2.0%*
*Likelihood d_1_/d_3_*	*6/1.4%*	*19/3.5%*	*2/0.5%*	*1/0.2%*	*28/1.4%*
Guessing	58/13.4%	54/9.9%	33/7.6%	36/6.4%	181/9.1%
Missing responses	54	61	48	18	181

### Results Concerning the Hypotheses

#### Hypotheses Concerning the Correct Denominator

The first hypothesis refers to differences in students’ abilities to indicate the correct basic set represented by *d*_1_ + *d*_3_. The results given by absolute and relative frequencies referring to each of the visualizations in brackets are shown in Table 4. The order of the visualization, that is, tree diagram – unit square in the first pair, and double tree diagram – 2 × 2-table in the second pair, represents the order in all hypotheses. Thus, in these hypotheses, we assume that the visualization on the right side of the two pairs is more efficient than the visualization on the left side.

**TABLE 4:** Frequencies for indicating *d*_1_ + *d*_3_ as denominator in a Bayesian situation.

**Visualization**	**Tree diagram**	**Unit square**	**Double-tree**	**2 × 2-table**	**Sum**
*d*_1_ + *d*_3_ indicated	256 (59%)	341 (62%)	304 (70%)	448 (79%)	1349 (68%)
*d*_1_ + *d*_3_ not indicated	178 (41%)	206 (38%)	128 (30%)	118 (21%)	630 (32%)

A χ^2^-test for independence indicating *d*_1_ + *d*_3_ did not produce a significant difference between a tree diagram and unit square (*df* = 1, χ^2^ = 2.91, *p* = 0.088). By contrast, the difference between a double tree diagram and 2 × 2-table was significant (*df* = 1, χ^2^ = 10.17, *p* < 0.05), with a small effect (odds ratio: 1.60; Cohen’s *d* = 0.20). Thus, hypothesis 1 was not confirmed, since the difference between a tree diagram and unit square was less pronounced than expected. By contrast, we found an unexpected difference between the double tree diagram and 2 × 2-table.

In an exploratory way, we also tested post-hoc the difference between visualizations regarding pairs of visualizations that differ in terms of the numerical information. Since there were four further pairs of visualizations with different numerical information, we ran χ^2^-tests using the Bonferroni-correction. In this case, the difference between a unit square and double tree diagram was significant (*p*^∗^ = 4*p* < 0.05, Cohen’s *d* = 0.17). The difference between a unit square and 2 × 2-table was highly significant (*p*^∗^ = 4*p* < 0.001), with a medium effect (Cohen’s *d* = 0.37). Finally, the difference between a tree diagram and both a double-tree diagram and 2 × 2-table was highly significant (*p*^∗^ < 0.001), with a nearly medium effect: Cohen’s *d* being between 0.24 and 0.45.

Hypothesis 1.1 refers to applying the Bayesian strategy restricted to those students who indicates *d*_1_ + *d*_3_ as denominator. In a subordinated hypothesis 1.1.1, we explored further if there was a dependency of the visualization, and a tendency to use *h*_1_ as numerator given the correct denominator *d*_1_ + *d*_3_. Due to the difference in the Bayesian situations, we involved only two Bayesian situations with *h_1_ < d_1_* + *d*_3_ for hypothesis 1.1.1. The related results for both hypotheses (1.1 and 1.1.1) are shown in Tables 5, 6.

**TABLE 5:** Frequencies for indicating the correct numerator when *d*_1_ + *d*_3_ is given as correct denominator in a Bayesian situation.

**Visualization (*d*_1_ + *d*_3_ indicated)**	**Tree diagram**	**Unit square**	**Double-tree**	**2 × 2-table**	**Sum**
*d*_1_ as numerator	162 (63%)	312 (92%)	238 (78%)	410 (92%)	1122 (83%)
not *d*_1_ as numerator	94 (37%)	29 (8%)	66 (22%)	38 (8%)	227 (17%)

**TABLE 6:** Frequencies for indicating *h*_1_ as numerator when *d*_1_ + *d*_3_ is given as correct denominator in a Bayesian situation.

**Visualization (*d*_1_ + *d*_3_ indicated), only cases with *h*_1_/(*d*_1_ + *d*_3_) < 1)**	**Tree diagram**	**Unit square**	**Double-tree**	**2 × 2-table**	**Sum**
*h*_1_ used as numerator	75 (50%)	23 (12%)	48 (28%)	29 (13%)	175 (24%)
*h*_1_ is not used as numerator	73 (50%)	176 (88%)	121 (72%)	197 (87%)	567 (76%)

The visualization seems to have a strong impact on the ability to correctly combine *d*_1_ + *d*_3_ and the correct numerator *d*_1_. A χ^2^-test found a highly significant difference between a tree diagram and a unit square (*df* = 1, χ^2^ = 71.16, *p* < 0.001), with a nearly high effect (odds ratio 6.2; *d* = 0.72). Also, the difference between a double tree diagram and 2 × 2-table was highly significant (*df* = 1, χ^2^ = 26.59, *p* < 0.001), with a medium effect (odds ratio 3.0; *d* = 0.38). For this reason, hypothesis 1.1 was confirmed.

Moreover, the difference between the tree diagram and both a double-tree diagram and 2 × 2-table was highly significant (*p*^∗^ = 4*p* < 0.001). The odds ratios were between 2.1 and 6.3, and Cohen’s *d* showed a medium effect for the double-tree diagram (*d* = 0.33), and a nearly high effect for the 2 × 2-table (*d* = 0.72). Finally, the difference between a double-tree diagram and a unit square was highly significant (*p*^∗^ = 4*p* < 0.001; *d* = 0.38). This means that both tree diagrams seem to hinder identification of *d*_1_ as numerator of the correct solution if the correct basic set is identified. This is also apparent in the comparison of a double tree diagram and unit square, although a double tree diagram provides more numerical information than a unit square.

For hypothesis 1.1.1, a χ^2^-test provided a highly significant result (*df* = 1, χ^2^ = 64.09, *p* < 0.001) concerning the difference between a tree diagram and unit square, with a high effect (odds ratio: 7.0; *d* = 0.93). The visualization strongly impacted the pre-Bayes strategy when *d*_1_ + *d*_3_ was identified as correct denominator. Further, the difference between a double-tree diagram and 2 × 2-table was highly significant (*df* = 1, χ^2^ = 14.94, *p* < 0.001), with a medium effect (*d* = 0.39). Thus, hypothesis 1.1.1 was confirmed. Both tree diagrams seem to trigger people to choose a node in the hierarchy of tree diagrams for identifying an adequate numerator.

Again, the difference between a tree diagram and both a double-tree diagram and 2 × 2-table was highly significant (*p*^∗^ = 4*p* < 0.001). The effect sizes varied concerning the odds ratio between 2.6 and 7.3, while Cohen’s d implied an at least medium effect (*d* = 0.46 for double-tree, and 0.88 for a 2 × 2-table). Moreover, the difference between a double-tree diagram and a unit square was highly significant (*p*^∗^ = 6*p* < 0.001; *d* = 0.39), although a double tree diagram provides more numerical information than a unit square.

#### Hypotheses Concerning the Correct Numerator

For testing Hypothesis 2, we analyzed the two pairs of visualizations concerning the use of the correct numerator *d*_1_. The related results are shown in Table 7.

**TABLE 7:** Frequencies for indicating d_1_ as the correct numerator in a Bayesian situation.

**Visualization**	**Tree diagram**	**Unit square**	**Double-tree**	**2 × 2-table**	**Sum**
d_1_ as numerator	268 (62%)	449 (82%)	320 (74%)	493 (87%)	1530 (77%)
Other numerator	166 (38%)	98 (18%)	112 (26%)	73 (13%)	464 (23%)

The ability to identify the correct numerator in a Bayesian situation was highly impacted by the visualization. The difference between a tree diagram and unit square was highly significant (*df* = 1, χ^2^ = 50.87, *p* < 0.001), with a medium effect (odds ratio: 2.8; *d* = 0.46). Further, the difference between the double-tree diagram and 2 × 2-table was significant (*df* = 1, χ^2^ = 27.54, *p* < 0.001), with a medium effect (*d* > 0.33). Thus, hypothesis 2 was confirmed. The tree diagrams seem to systematically hinder people to identify the correct numerator. Again, the difference between a tree diagram and both a double-tree diagram and 2 × 2-table was highly significant (*p*^∗^ = 4*p* < 0.001). Moreover, the difference between a double-tree diagram and unit square was significant (*p*^∗^ = 4*p* < 0.05; *d* = 0.17), although a double tree diagram provides more numerical information than a unit square.

Hypothesis 2.1 refers to the amount of correct solutions with the indication of *d*_1_ as correct numerator. In a pair of subordinated hypotheses (2.1.1 and 2.1.2), we further explored the dependency of the visualizations and tendency to use *h*_1_ or *n* as denominator given the correct numerator *d*_1_. The results concerning these three hypotheses are shown in Table 8.

**TABLE 8:** Frequencies for indicating the correct solution, *n* as denominator, or *h*_1_ as denominator, given *d*_1_ as the correct numerator in a Bayesian situation.

**Visualization (d_1_ as numerator)**	**Tree diagram**	**Unit square**	**Double-tree**	**2 × 2-table**	**Sum**
*d*_1_ + *d*_3_ as denominator	162 (60%)	312 (70%)	238 (74%)	410 (83%)	1122 (73%)
not *d*_1_ + *d*_3_ as denominator	106 (40%)	137 (30%)	82 (26%)	83 (17%)	408 (37%)
*h*_1_ used as denominator	69 (26%)	64 (14%)	35 (11%)	56 (11%)	224 (15%)
Other denominator	199 (74%)	385 (86%)	285 (89%)	437 (89%)	1271 (85%)
*n* used as denominator	22 (8%)	44 (10%)	30 (9%)	18 (4%)	114 (7%)
Other denominator	246 (92%)	405 (90%)	290 (91%)	475 (96%)	1416 (93%)

For hypothesis 2.1.1, a χ^2^-test showed that the dependency of indicating *h*_1_ as denominator given *d*_1_ as correct numerator and the visualization was significant. The difference between a tree diagram and a unit square was highly significant (*df* = 1, χ^2^ = 14.67, *p* < 0.001), with a nearly medium effect (odds ratio: 2.1, Cohen’s *d* = 0.29). By contrast, the difference between a double tree diagram and a 2 × 2-table was not significant. Thus, hypothesis 2.1.1 was partly confirmed for hypothesis 2.1.1a).

Further, the difference between a tree diagram and a double-tree diagram and 2 × 2-table was highly significant (*p*^∗^ = 4*p* < 0.01), with a medium effect (*d* = 0.39 and 0.37).

The tendency to identify the incorrect denominator *n* combined with the correct numerator *d*_1_ was partly impacted by the visualization. The difference between a tree diagram and unit square was not significant. By contrast, the difference between a double-tree diagram and 2 × 2-table was significant (*df* = 1, χ^2^ = 11.44, *p* < 0.001), with a small effect (odds ratio: 2.7; *d* = 0.23). Thus, hypothesis 2.1.1 was partly confirmed for hypothesis 2.1.1b). Moreover, the difference between the three visualizations, that is a tree diagram, a double tree diagram and a unit square, and a 2 × 2-table was significant with a small effect.

#### Hypothesis Concerning the Specific Proportion of Numerator and Denominator

Finally, we tested hypothesis 3. Table 9 shows the results for both scenarios, *d*_1_ + *d*_3_ > *h*_1_, and *d*_1_ + *d*_3_ < *h*_1_. The relative frequency is based on the number of solutions for each visualization in each of the two scenarios.

**TABLE 9:** Pre-Bayes strategy for situations with *d*_1_ + *d*_3_ > *h*_1_ and with *d*_1_ + *d*_3_ < *h*_1_.

**Visualization**	**Tree diagram**	**Unit square**	**Double-tree**	**2 × 2-table**	**Sum**
*h*_1_/(*d*_1_ + *d*_3_) > 1: pre-Bayes	19 (9%)	3 (1%)	16 (7%)	5 (2%)	43 (4%)
*h*_1_/(*d*_1_ + *d*_3_) > 1: no pre-Bayes	205 (91%)	268 (99%)	209 (93%)	265 (98%)	947 (96%)
*h*_1_/(*d*_1_ + *d*_3_) < 1: pre-Bayes	75 (38%)	23 (9%)	48 (24%)	29 (10%)	175 (19%)
*h*_1_/(*d*_1_ + *d*_3_) < 1: no pre-Bayes	123 (62%)	232 (91%)	153 (76%)	253 (90%)	761 (81%)

The difference concerning the sum of the four visualizations produced a highly significant result (*df* = 1, χ^2^ = 98.75, *p* < 0.001). The highly significant difference appeared for each of the visualizations as well. Thus, the context represented by a specific proportion of the numerator and denominator has a significant impact on the pre-Bayes strategy in Bayesian situations.

### Use of the Strategies Described in the Literature

Additionally, we analyzed differences between the visualizations referring to the erroneous strategies reported in Table 1. Table 10 indicates if a visualization in the first column shows a significantly higher amount of people showing a specific strategy. We do not regard the accumulation of hypotheses in this case. For this reason, the results must be interpreted carefully. Referring to the pre-Bayes strategy, we again restricted the analysis to two tasks.

**TABLE 10:** Differences among the visualizations referring to strategies shown in Table 1 based on the entirety of students’ answers.

	**Tree diagram**	**Unit square**		**Double-tree**		**2 × 2-table**	
Tree diagram		*p* < 0.001:	pre-Bayes	*p* < 0.001:	rep. think.	*p* < 0.01:	pre-Bayes
		*p* < 0.05:	evid. only,	*p* < 0.001:	pre-Bayes		evid. only
						*p* < 0.01:	rep. think. conserv.
Double-tree		*p* < 0.001:	pre-Bayes			*p* < 0.001:	pre-Bayes
						*p* < 0.01:	joint occ.
2 × 2-table							
Unit square						*p* < 0.001:	joint occ.
						*p* < 0.01:	conserv.

## Discussion

The main aim of this paper was to contribute to the field of facilitating Bayesian reasoning by focusing on people who fail to use the correct strategy in a Bayesian situation, even though the statistical information is given by natural frequencies and visualization. We focused on two pairs of visualizations. According to Khan et al. (2015), the visualizations within a pair provide mostly the same numerical information but differ in style, that is, a branch style and a nested style, and further differ in graphical transparency. Visualizations between the two pairs differ in at least the numerical information and, thus, in numerical transparency. To investigate people’s erroneous strategies, we differentiated between identifying the correct basic set and the correct subset of the nested-sets structure in a Bayesian situation. We realized this approach by asking people to respond with a fraction. This allowed us to analyze erroneous responses concerning the denominator and the numerator. However, since other studies use a single step frequency version for a response, findings in these studies must be compared with caution with our results. Our results provide substantial evidence that people’s strategies in Bayesian situations are strongly dependent on different visualizations. Thus, a specific visualization hinders or facilitates identification of the relevant basic set *D* represented by the denominator *d*_1_ + *d*_3_, and the relevant subset *H* ∩ *D* represented by the numerator *d*_1_.

We first analyzed different strategies regarding identification of the correct basic set *D* (hypothesis 1). We found that numerical transparency has the main impact. We did not find significant differences within the two pairs of visualization, that is, between a tree diagram and a unit square, and between a double tree diagram and a 2 × 2-table. By contrast, but as expected, the difference between the two visualizations that provide the relevant subset (*D*) numerically (double tree diagram and 2 × 2-table) and the two visualizations that do not provide this numerical information (tree diagram and unit square) is significant. Against expectations, a unit square was not found to be more effective for identification of the correct basic set in a Bayesian situation compared to the tree diagram. This was an unexpected result, since the mentioned partition of *D* is transparent in the unit square, but not in a tree diagram. Regarding a differentiation between the relevant basic set (denominator) and subset (numerator), our result contributes to the discussion of transparency of the nested-sets relation in a Bayesian situation by focusing on the visualizations’ characteristics (cf. Sloman et al., 2003).

In subordinated hypotheses, the students’ responses were restricted to those in which the basic set *D* was correctly identified. The correct identification of the basic set in visualizations representing a nested style (unit square, 2 × 2 table, cf. Khan et al., 2015) almost always goes along with the use of a Bayesian strategy: 92% of the responses with the correct basic set show the correct Bayesian strategy. Students who use a visualization representing the branch style (tree diagrams, cf. Khan et al., 2015) and who identified the correct basic set use the correct Bayesian strategy to a lesser extent: only 78% of the students using a double-tree diagram and 63% of the students using a tree diagram used the Bayesian strategy, although they were able to identify the correct basic set *D*. More specifically, our results show that both tree diagrams trigger the identification of *H* as a relevant subset. We expected a difference between a tree diagram and unit square since the relation between the basic set *D* and the subset *H* ∩ *D* is not visualized in the hierarchy of the tree diagram and is therefore not transparent. However, a study by Bruckmaier et al. (2019) suggests that people tend to search for a set-subset relation in the hierarchy of a tree diagram. For this reason, the tree diagram hinders use of the Bayesian strategy compared to other visualizations such as unit square, since a tree diagram obscures the nested-sets structure of a Bayesian situation. We did not expect the difference between a double tree diagram and 2 × 2-table, and even between a double tree diagram and unit square. This result provides evidence that a graphical transparency is effective beyond a numerical transparency. A possible, but speculative interpretation of this result, is that the two hierarchies in a double-tree diagram partly trigger people to identify the basic set *D* with its subsets *H*∩*D* and $\bar{H}\cap D$. If this is the case, the challenge is the same as for a tree diagram, that is, to identify a subset of $\left(H\cap D\right)\cup \left(\bar{H}\cap D\right)$ in the (first) hierarchy of a double-tree diagram. However, this interpretation should be investigated in future research.

A second analysis started with identification of the correct subset *H* ∩ *D*. As expected, the result indicated that identifying the correct subset *H* ∩ *D* is strongly impacted by the visualization. Thus, a 2 × 2-table and a unit square are more effective for identifying the correct subset in a Bayesian situation, although the subset is given by a node in both tree diagrams. We interpret this result by the transparency of the subset *H* ∩ *D* as an intersection set. Thus, a field within a 2 × 2-table or unit square implies representing an intersection of sets represented by the two sides of the field. By contrast, the hierarchical path of both tree diagrams makes the property of *H* ∩ *D* as intersection set not transparent to the same extent. This result agrees with the findings of Bruckmaier et al. (2019) regarding the analysis of people’s ability to identify conjoint probabilities in a tree diagram and a 2 × 2-table. Our results concur with the findings of Binder et al. (2020), who found that a 2 × 2-table facilitates identifying conjoint events compared to a double tree diagram. The result also goes along with our own finding in Böcherer-Linder et al. (2018) that people’s performance can be increased by making the subset *H* ∩ *D* as intersection set, graphically transparent.

The results for hypothesis 2.1 are similar to the results for hypothesis 1: it is easier to identify the correct basic set (*D*) in the 2 × 2-table and the double-tree diagram, for which the basic set is explicitly given (numerical transparency), than in a unit square and a tree diagram. In contrast to the results concerning hypothesis 1, it is easier to identify the basic set in a unit square than in a tree diagram, for which the basic set *D* is not transparent. The result concerning hypothesis 2.1.1 strengthens the assumption that a visualization’s hierarchy may be a disadvantage when dealing with Bayesian situations. Thus, a unit square was found to be significantly more effective compared to a tree diagram in order to avoid the representative thinking strategy (*d_1_/h_1_*), when the correct subset is identified. Also, a double tree diagram is more effective in avoiding this strategy than a tree diagram. We interpret this result considering the property of the double-tree diagram to propose two possibilities for identifying the correct basic set in the hierarchy of the tree, that is, the nodes representing the frequencies of *h*_1_ and of *d*_1_ + *d*_3_, whereas the tree diagram proposes only the node representing *h*_1_.

With hypothesis 3, we regarded the influence of the Bayesian situation’s context that is given by the two scenarios *h_1_/(d_1_* + *d_3_) <* 1 and *h_1_/(d_1_* + *d_3_) >* 1. The difference in the Bayesian situations strongly impacts the amount of responses showing the pre-Bayes strategy. Thus, whereas the pre-Bayes strategy is of minor importance if *h_1_/(d_1_* + *d_3_) >* 1, it is an often used strategy if *h_1_/(d_1_* + *d_3_) <* 1. This finding is apparent for each of the four visualizations. Accordingly, research either yielded the pre-Bayes strategy (Zhu and Gigerenzer, 2006), or not (Bruckmaier et al., 2019).

The strategies described so far in literature (Table 1) are mostly dependent on visualization. The most prominent strategy is the correct Bayesian strategy that people used in between 37.3% (tree diagram) to 72.4% (2 × 2-table) of the cases. Thus, visualization was again found to strongly impact people’s performance in Bayesian situations. Nevertheless, there are some studies that did not find a facilitating effect of visualization (e.g., icon arrays in Sirota et al., 2014; Euler-diagrams in Brase, 2009). For this reason, and congruent with the research of Binder et al. (2015) and Binder et al. (2020), we found that visualization in combination with natural frequencies strongly impacted people’s performance in Bayesian situations. We have analyzed differences in people’s performance concerning visualization before (Böcherer-Linder and Eichler, 2019). In this paper, erroneous strategies are of particular importance. In this regard, our findings replicate the results of Zhu and Gigerenzer (2006) with respect to the existence of the main strategies (Table 1). However, the work of Zhu and Gigerenzer (2006) is expanded through our research, since the strategies are described as being dependent on different visualizations. Further, we contribute to the analysis of erroneous strategies by a differentiated focus on the basic set *D* and the subset *H*∩*D*. In our results, the most prominent erroneous strategy was the pre-Bayes strategy. As outlined above, this strategy depends on the situation and visualization. Particularly, a unit square and a 2 × 2-table are more effective at avoiding the pre-Bayes strategy compared to both tree diagrams. The second significant erroneous strategy is the representative thinking strategy. The representative thinking strategy is highly dependent on a visualization, and seems to be triggered especially by a tree diagram and its hierarchy as outlined in hypothesis 2.1.2.

The other systematic erroneous strategies are of less importance if all visualizations are considered. However, for a part of the visualizations, specific strategies are of importance. For example, since it seems to be easy to identify the correct subset (numerator) in a Bayesian situation when a unit square is used (Table 7), to identify in addition the correct basic set (denominator) seems to be a bigger challenge and yields a considerable amount of joint occurrence strategy (*d_1_/n*) and likelihood strategy (*d_1_/d_3_*).

Our results contribute to existing research on Bayesian reasoning, particularly to research concerning people’s erroneous strategies in Bayesian situations. Moreover, our results have implications for mathematics education, specifically the teaching and learning of conditional probabilities and Bayes’ formula. Due to the relevance of these subjects for inferential judgements in situations of uncertainty in real life and the relevance of these subjects for learning probability in school, understanding how to facilitate Bayesian reasoning and avoid erroneous strategies is important. A striking result concerns a property of a tree diagram compared to the three other visualizations that differ in graphical transparency (unit square), numerical transparency (double tree diagram), or graphical and numerical transparency (2 × 2-table): a tree diagram seems to trigger the identification of an erroneous basic set and, in particular, an erroneous subset in a Bayesian situation. This result is interesting, since the tree diagram is one of the most common visualizations of Bayesian situations (e.g., Utts and Heckard, 2015). For this reason, favoring the tree diagram as a visualization to improve Bayesian reasoning may be questioned.

Further, our results can be used to improve trainings of Bayesian reasoning that are based on a double-tree diagram (Wassner, 2004) or a unit square (Talboy and Schneider, 2017). When using a double-tree diagram, a specific focus must be put on identifying the correct subset *H*∩*D*, and emphasizing the related node as representing the intersection set *H*∩*D* that allows for the set inclusion (*H*∩*D*)⊆*D*. When using a unit square, our results imply that a specific focus must be put on identification of the correct basic set, since most of the students found a correct strategy based on this identification. We assume that a brief training focusing on the mentioned aspects can result in a considerable impact on the facilitating effect of a double-tree diagram and a unit square.

A 2 × 2-table seems to appear as an optimal visualization of a Bayesian situation. Although this statement is clearly supported by the results of this study and is also implied by other studies (Binder et al., 2015; Bruckmaier et al., 2019; Böcherer-Linder and Eichler, 2019), this statement must be interpreted carefully. Firstly, for the students in our study, the 2 × 2-table was a very familiar visualization. With our study design, we were not able to estimate the impact of this fact. However, the results regarding the tree diagram that was also very familiar to the students provided evidence that familiarity is not as important for a facilitating effect as other characteristics of a visualization. Furthermore, we follow Bruckmaier et al. (2019), stating that a 2 × 2-table is restricted to Bayesian situations that are given in a natural frequency format. If a Bayesian situation is given in a probability format with *P*(*H*),*P*(*D*|*H*) and $P\,\left(D|\bar{H}\right)$, the conditional probabilities cannot be visualized by a 2 × 2-table. Thus, to draw a 2 × 2-table based on this information in the probability format necessitates computing the information in a 2 × 2-table. This is not necessary for the other visualizations, that is, a tree diagram, a double-tree diagram, or a unit square. For this reason, we assume that the facilitating effect of a 2 × 2-table is restricted to situations in which the statistical information of a Bayesian situation is entirely given in a natural frequency format.

Finally, an open question remains about the effect of visualizations on people’s erroneous strategies when they have been trained in using visualizations before. This research may lead to further enhancement on the facilitating effect of visualization and its impact on people’s strategies in Bayesian situations.

## Conclusion

We illustrated that people’s strategies in Bayesian situations depend strongly on specific visualizations of the statistical information in these situations. Different visualizations trigger specific ways of identifying a basic set and related subset in Bayesian situations. Although each of the visualizations in our research, that is, a tree diagram, a unit square, a double-tree diagram, and a 2 × 2-table were found to improve people’s performance in Bayesian situations, a tree diagram triggers significantly more erroneous strategies in comparison to the other three visualizations. The differences may be explained by a numerical transparency. In our research, the numerical transparency is implied if the basic set of a Bayesian situation is explicitly given by a field or a node. However, beyond the amount of numerical information, making the nested-sets structure of a Bayesian situation graphically transparent seems to help avoid erroneous strategies. In our research, the nested-sets structure of a Bayesian situation was in particular graphically transparent when a subset could be clearly identified as an intersection set. Our findings contribute to the debate about beneficial graphical properties of visual representations of statistical information in Bayesian situations, and serve as an empirical foundation in mathematics education for designing interventions to improve Bayesian reasoning.

## Data Availability Statement

The datasets generated for this study are available in a free accessible repository (https://osf.io/w64n5/).

## Ethics Statement

The studies involving human participants were reviewed and approved by Geschäftsstelle der zentralen Ethikkommission der Universität Kassel Mönchebergstr. 19 34125 Kassel Germany, E-Mail: ethikkommission@uni-kassel.de. Written informed consent for participation was not required for this study in accordance with the national legislation and the institutional requirements.

## Author Contributions

All authors listed have made a substantial, direct and intellectual contribution to the work, and approved it for publication.

## Conflict of Interest

The authors declare that the research was conducted in the absence of any commercial or financial relationships that could be construed as a potential conflict of interest.

## Footnotes

1 https://osf.io/w64n5/
